# Psychological Detachment in the Relationship between Job Stressors and Strain

**DOI:** 10.3390/bs3030418

**Published:** 2013-07-23

**Authors:** My Safstrom, Terry Hartig

**Affiliations:** 1 Department of Psychology, Uppsala University, Box 1225, Uppsala SE-75142, Sweden; 2 Institute for Housing and Urban Research, Uppsala University, Box 514, Uppsala SE-75120, Sweden; E-Mail: terry.hartig@ibf.uu.se

**Keywords:** post-work recovery, psychological detachment, job demands, restorative environments, Sweden

## Abstract

We investigated the mediating *versus* moderating role of psychological detachment in the relationship between job stressors and psychological strain. Our sample consisted of 173 university students invested in challenging programs of advanced professional studies, who could find it difficult to detach from work. Hierarchical regression analyses of cross-sectional survey data affirmed the role of psychological detachment as a mediator in the relationship between job demands and perceived stress. Detachment also mediated the relationship between job demands and satisfaction with life, although the association disappeared when controlling for negative affectivity. Detachment did not mediate relationships between job demands and cognitive failures. Psychological detachment did not moderate any of the investigated relationships. The study contributes to a view of psychological detachment as less subject to individual differences than to the imposition of stressors in the given context.

## 1. Introduction

Recovery from work is necessary for workers to avoid chronic stress. According to Meijman and Mulder’s effort-recovery model, recovery occurs when work demands no longer strain the individual’s resources [1]. In accordance with this view, mentally distancing oneself or detaching from job stressors is assumed to contribute to recovery (e.g., [1,2]). Psychological detachment occurs when the individual is not occupied with work-related thoughts, tasks or emotions, but instead, disengages psychologically from work [3].

**Figure 1:**
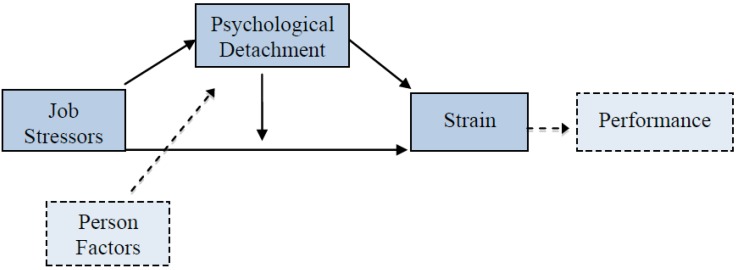
Sonnentag’s [4] stressor-detachment model. Psychological detachment as mediator and moderator in the relationship between job stressors and strain (solid boxes refer to main concepts of the model; dotted boxes refer to concepts contributing and resulting from psychological detachment and strain).

Reprinted with permission from [4].

Sonnentag [4] has proposed a model of how psychological detachment affects and is affected by other key variables. In her model, psychological detachment from work is described as both a mediator and a potential moderator in the relationship between job stressors and strain (see Figure 1). When psychological detachment from work is low, no recovery can take place, and strain reactions will continue. In other words, detachment works as a mediator. Psychological detachment can also work as a moderator, in which case, the relationship between job stressors and strain is stronger when detachment is low and *vice versa*, as, for instance, depending on an individual’s own capacity to detach from work related thoughts and actions.

The direct relationships in the model have been empirically substantiated in multiple studies. The main relationship between job stressors and strain is well established [5,6]. Studies have also shown that different kinds of job stressors are associated with psychological detachment. For instance, quantitative work demands, job ambiguity, hours of overtime and role conflict have all been shown to correlate negatively with detachment [7,8,9]. Finally, studies have shown that successful psychological detachment from work appears to reduce psychological strain and enhance well-being, as reflected in lower job exhaustion, emotional exhaustion and need for recovery [7,8], fewer depressive symptoms and health problems [8] and higher satisfaction with life [8,10].

Although the empirical evidence just mentioned is consistent with mediation, it seems the mediation model has not often been formally tested. To our knowledge, only one published study has involved a formal test of mediation [11]. Psychological detachment was there shown to partially mediate the relationship between job stressors (workload and emotional dissonance at work) and the individuals’ need for recovery. 

The model in Figure 1 also depicts psychological detachment as a potential buffer of the negative impact of job stressors on strain reactions. This possibility has received more attention than the mediation model (e.g., [7,12,13,14]). One can, however, question the model fit in these studies, since all, except one, show small contributions (≤ 2%) to explained variance when the moderation term is added.

Siltaloppi *et al*. [7] showed that detachment moderated the relationship between a lack of job control and an increased need for recovery, but the moderation term explained only 1% of additional variance (*p* < 0.01; N = 485). Moreno-Jiménez, Rodríguez-Muñoz *et al*. [12] found that detachment moderated the relationship between bullying at work and psychological strain. Here, the moderator term explained 2% additional variance (*p* < 0.05; N = 511). Sonnentag, Binnewies *et al*. [13] found 1.1% additional variance explained when they included detachment as a moderator of the negative relationship between quantitative job demands and work engagement (*p* < 0.05; N = 309). They also found that detachment as a moderator explained 0.6% of variance in psychosomatic complaints (*p* < 0.05; N = 309) in a model with quantitative job demands as the job stressor. The one exception in this set is the study by Moreno-Jiménez, Mayo, *et al*. [14], who found detachment to interact with work-family conflict (*i.e*., work interfering with family responsibilities) in predicting psychological strain (*p* < 0.01; N = 128). Detachment was not the only moderator entered at this step, however. The other moderator, verbal expression of emotion, was also a significant contributor to explanation. Thus, the relatively large amount of additional variance in strain explained at this step in the analysis (6%) cannot be attributed to detachment alone. 

It is problematic to regard psychological detachment as both a mediator and a moderator. Sonnentag herself has called this a dilemma [4] and points to the possibility that psychological detachment is, under specific circumstances, a mediator and, under others, a moderator ([4], p. 264). This openness seems, however, not to have characterized the approach in the four aforementioned studies of moderation. None of the articles mentioned mediation as a possible alternative. However, if one takes a closer look at the results of Sonnentag, Binnewies *et al*. [13] and Moreno-Jiménez, Mayo *et al*. [14], one finds that the stressors are significantly related to detachment (r = −0.21 to −0.37), a condition that is required by the logic of mediation, but which is not desirable in tests of moderation models [15]. Furthermore, the model tested by Moreno-Jiménez, Mayo *et al*. [14] differs from the models in the other three studies, since the stressor in focus, work-family conflict, in itself incorporates a detachment problem. By implicitly placing the conflict in the home, assumed in this literature to be a key setting for recovery from work, the model builds the mediating role of detachment into the stressor itself. This combination has implications for the results. Work-family conflict was a significant predictor of psychological strain when first entered into the analysis; however, its coefficient became insignificant when psychological detachment was subsequently entered into the model. Importantly, similar evidence of overlap with detachment was not found for family-work conflict (*i.e*., family responsibilities interfering with work), nor did family-work conflict interact with detachment in predicting psychological strain. The characteristics of the putative stressor, work-family conflict, could be another reason for the relatively greater strength of the interaction term in this particular study, as mentioned above.

A model with psychological detachment as a moderator essentially treats it as an individual difference variable. Such a model may be allowed little explanatory power when job stressors themselves can strongly affect the individuals’ level of detachment. Herein may lie the answer to the dilemma posed by Sonnentag. Consider psychological detachment as a coping strategy, much like social withdrawal. What happens in the circumstances where the strategy cannot be applied? What if the context is such that one cannot detach? Bullying at work or a lack of job control might not trouble a person at home if he or she is an individual with a high capability for detachment; however, a heavy workload may result in a perceived need to take work home and, thus, disrupt the possibility for detachment there, regardless of the person’s detachment capability. Conceivably, when the context of the job stressors affects the possibility for psychological detachment to occur, detachment works as a mediator. Under other circumstances, the ability to detach acts as an individual difference variable, and a moderation model is more appropriate. The discriminating results for work-family *versus* family-work conflict reported by Moreno-Jiménez, Mayo *et al*. [14] are instructive in this regard. 

In this study, we investigate the mediating and moderating role of psychological detachment in the relationship between job stressors and psychological strain. To capture variation in stressors, we used a measure of job demands that combines three components: quantitative demands, or the working pressure that the individual is under; decision demands, or the need for attention and focus in a decision making process; and learning demands, or the difficulty level of tasks, as well as the individual’s need to acquire new knowledge and skills to complete them [16].

We have also used three different outcome measures to cover different areas of psychological strain and well-being. We have used a well-established measure of perceived chronic stress [17], due to the negative impact that chronic stress has on health [18]. We have also used a measure of minor, common, cognitive mistakes [19] to assess a deficit in effective functioning. Lastly, we have used a measure of life satisfaction [20] to gain a subjective view of the respondent’s overall well-being. Our analyses of all three outcomes include characteristics of the individual and work context as additional covariates, as described below.

Quantitative demands have in several previous studies been strongly negatively related to psychological detachment (*r* = −0.46 to −0.49) [7,8,9]. In this study, we therefore hypothesize that psychological detachment from work will mediate an association between job demands and psychological strain. Psychological detachment is not expected to be a moderator of the same relationship, given that it is expected to have a strong relationship with demands and that in the present context work demands are assumed to follow the participants across settings, including the home.

## 2. Experimental Section

### 2.1. Participants and Procedures

Our sample consisted of students who were enrolled in one of several professional programs at Uppsala University in Sweden (nursing, engineering, law and counseling psychology). This occupational model was selected, because the students are working full-time with mentally demanding tasks, and their working hours are adaptable to their current workload, so that they can perform a variety of tasks at a given time and are under pressure to do so. This situation resembles contemporary employment conditions outside of higher education, with dissipating boundaries between work and leisure [21,22]. To participate, the students had to have begun at least their second year of study. This increased the likelihood that a high level of motivation and effort would be found among the participants, that they would be invested in their studies and that they would have a persistent need for recovery. Another inclusion criterion was not having children. A similar criterion has been applied in previous studies [3,9] to eliminate confounding by the demands of childcare and conflict between work and family demands. 

The study was conducted in accordance with the current ethical rules of Uppsala University. A total of 646 students were registered in the four programs. During a regular lecture, the students were informed of the purpose of the study and asked whether they wanted to participate. Participation was voluntary, but a chance to win a movie ticket was offered as an incentive. Those who volunteered were given a survey to fill in on location after the lecture was finished. The total number of registered students can be considered the maximum number of students asked, but not all students attended all lectures. Of these 646 students, 174 chose to participate (27%), one of which was excluded, due to an incomplete survey form. This left 173 participants with usable data. They ranged in age from 20 to 30 years (M = 23.2, SD = 2.1), and 66 % of them were women. 

### 2.2. Measures

#### 2.2.1. Job Demands

Three subscales from the General Nordic Questionnaire for Psychological and Social Factors at Work (QPS_Nordic_) were used to assess different components of job demands imposed by the students’ academic work during the last month [16]. Quantitative demands consists of four items (e.g., “Do you have too much to do?”), decision demands of three items (e.g., “Does your work require quick decisions?”) and learning demands of three items (e.g., “Are your work tasks too difficult for you?”), all of which are scored on a 5-point scale ranging from 1 (very seldom or never) to 5 (very often or always). We combined the subscales into a single measure of job demands to improve measurement reliability; the scale had acceptable internal consistency (Cronbach’s alpha = 0.77). Use of the single measure for job demands also simplified the analyses. Our job demand measure has been shown to predict emotional exhaustion and stress symptoms, such as nervousness and sleep difficulties in samples of Nordic workers [16].

#### 2.2.2. Stress

The Perceived Stress Scale (PSS; [17]) consists of ten items and measures the degree to which one perceives one’s circumstances as persistently uncontrollable, unpredictable and overloading (e.g., “In the last month, how often have you felt nervous and stressed?”). Items are scored on a 5-point scale from 0 (never) to 4 (very often). Cronbach’s alpha in the present sample was 0.87. Scores on the PSS have been shown to predict general health, as well as speed of wound healing and susceptibility to the common cold, due to impaired immune function (e.g., [23,24]).

#### 2.2.3. Cognitive Failures

The Cognitive Failures Questionnaire (CFQ; [19]) was used to assess the participants’ degree of persistent cognitive fatigue. It consists of 25 items (e.g., “Do you drop things?”; “Do you find you forget appointments?”), which are scored on a 5-point scale from 0 (never) to 4 (very often). The time frame for the scale ordinarily specifies the last six months, but it was adapted to ask only for the participant’s experience in the last month, so that it would not include the summer holidays. Cronbach’s alpha was 0.86. Studies have shown that cognitive failures are related to slow performance on focused attention tasks [25], as well as automobile and work accidents [26]. Broadbent *et al*. initially discussed the CFQ as a trait rather than a state measure [19]. Its status as a trait measure may, however, reflect on the stability of circumstances that are cognitively depleting rather than some cognitive limitation that is inherent in an individual.

#### 2.2.4. Satisfaction with Life

The Satisfaction with Life Scale (SWLS; [20]) was used to assess the participants’ current satisfaction with life. The measure consists of five items (e.g., “I am satisfied with my life”.), which are scored on a 7-point scale from 1 (strongly disagree) to 7 (strongly agree). Cronbach’s alpha in this study was 0.83. Studies have shown SWLS to be positively correlated with other scales measuring subjective well-being [20].

#### 2.2.5. Psychological Detachment

The Psychological Detachment scale from Sonnentag and Fritz’s [8] Recovery Experience Questionnaire was used to measure the participants’ degree of psychological detachment during the last month. Due to the time frame, it could thus be taken as an indication of individual difference, as well as a response to persistent demands bound to the given work context. The measure consists of four items (e.g., “During leisure time, I forget about work.”), which are scored on a 5-point scale from 1 (fully disagree) to 5 (fully agree). To avoid confusion, the term “work” was specified as “academic work”. For the purpose of this study, the participants were asked to answer the questions with regard to their leisure time, both during the week and during their weekends. However, due to a high correlation between the two versions, the answers were later joined in a single measure. Cronbach’s alpha was 0.88. Psychological detachment as measured with this scale has been shown to correlate negatively with several measures of psychological strain, such as depressive symptoms and health problems [8].

#### 2.2.6. Control Variables

Statistical models also included gender (male = 1, female = 2), age in years, study program (1 = nurse, 2 = engineer, 3 = lawyer during 4th semester, 4 = psychologist, 5 = lawyer during 5th semester; for the sake of parsimony, dichotomized into 1 = not law student, 2 = law student, those two categories that an ANOVA and subsequent *post hoc* tests indicated were relevant to the multivariate tests), hours per week spent on academic work during the weekdays and during weekends and whether or not the participants had a deadline in the next few days (1 = yes, 2 = no).

Negative affectivity could possibly explain relationships between predictors and outcomes [27]. Negative affectivity was measured with the negative affect scale from the Positive and Negative Affect Schedule [28], which consists of ten different affective adjectives (e.g., “upset”; “scared”). The participants were asked to what extent they felt like this in general. Answers were scored on a 5-point scale from 1 (very slightly or not at all) to 5 (extremely). Cronbach’s alpha was 0.83.

## 3. Results and Discussion

Table 1 shows means, standard deviations and zero-order correlations for all study variables. The strong correlation between program of study and whether or not the participant had a deadline in the next few days (r = −0.73) indicated a possible problem with multicollinearity. Deadline alone was, therefore, used as a covariate in the multivariate analyses, since it was thought to provide more specific information regarding current demands. 

### 3.1. Tests of Mediation

An initial regression analysis was conducted for each outcome measure using an SPSS macro published by Preacher and Hayes [29] to obtain a bias-corrected bootstrapped 95% confidence interval for the indirect effect of job demands as mediated by detachment. In addition to job demands, each analysis included gender, deadline and academic work on weekdays as covariates. In light of debate concerning whether or not to adjust for negative affectivity in job stress research (cf. [27]), the initial set of regressions did not include negative affectivity; however, we conducted an extra set of analyses that included it among the covariates. After first presenting the initial set of regressions, we note those few differences found with negative affectivity included in the model.

#### 3.1.1. Perceived Stress as the Outcome

Table 2 shows the results from the regression analyses of perceived stress and the other two outcomes. Of the control variables, only gender was a statistically significant predictor of perceived stress. In total, the model explained about 33% of the explained variance in that outcome. Both job demands and detachment predicted stress, and job demands also predicted detachment. This pattern of relationships suggests mediation [15]. The impression of mediation is affirmed by the confidence interval (CI) for the indirect effect, which did not include zero (95% CI = 0.0247 − 0.1749). The result indicates that a significant degree of mediation and is in line with the study hypothesis. Job demands remained a significant predictor after adjustment for detachment, so only partial mediation is indicated.

**Table 1:** Means, standard deviations and Pearson correlations for study variables.

	M	SD	1	2	3	4	5	6	7	8	9	10
1. Gender	---	---										
2. Program	---	---	0.07									
3. Deadline	---	---	0.02	−0.73 **								
4. Academic work weekdays	30.71	12.07	0.16 *	−0.20 **	−0.09							
5. Academic work weekends	6.21	3.88	−0.00	0.27 **	−0.16 *	0.46 **						
6. Negative affectivity	1.99	0.57	0.11	0.13	−0.15	0.03	0.19 *					
7. Job demands	3.16	0.60	0.04	0.44 **	−0.35 **	0.27 **	0.42 **	0.35 **				
8. Detachment	2.05	0.81	−0.09	−0.30 **	0.23 **	−0.35 **	−0.49 **	−0.27 **	−0.52 **			
9. Stress	1.82	0.66	0.29 **	0.26 **	−0.24 **	0.14	0.20 **	0.67 **	0.48 **	−0.41 **		
10. Cognitive failures	1.79	0.47	0.03	0.08	−0.07	0.09	0.12	0.54 **	0.36 **	−0.20 **	0.55 **	
11. Life satisfaction	4.83	1.14	0.15	−0.09	0.14	0.01	−0.12	−0.40 **	−0.21 **	0.21 **	−0.44 **	−0.37 **

* *p* < 0.05; ** *p* < 0.01.

**Table 2:** Results from regressions analyses with psychological detachment as a mediator of the relationships between job demands and outcome variables.

	Perceived Stress			Cognitive Failures			Satisfaction with Life		
	B	SE_B_	t	B	SE_B_	t	B	SE_B_	t
**Control variables **									
Gender	0.352	0.089	3.97 ***	0.008	0.072	0.114	0.407	0.179	2.278 *
Deadline	−0.108	0.089	−1.22	0.053	0.073	0.727	0.104	0.180	0.575
Acad. Work weekends	−0.011	0.013	−0.904	−0.007	0.010	−0.676	0.003	0.026	0.118
Direct effect ofJob Demands on Detachment	−0.476	0.095	−5.00 ***	−0.476	0.095	−5.00 ***	−0.476	0.095	−5.00 ***
Direct effect ofDetachment onoutcome	−0.177	0.066	−2.69 **	−0.018	0.054	−0.341	0.268	0.133	2.017 *
Total effect of Job Demands on outcome	0.472	0.082	5.75 ***	0.0312	0.066	4.756 ***	−0.332	0.164	−2.020 *
Effect of Job Demands on outcome after adjustment forDetachment	0.388	0.086	4.49 ***	0.304	0.071	4.298 ***	−0.205	0.175	−1.171
*Model summary*									
*R^2 ^*			0.337			0.135			0.097
*(R^2^adj)*			0.317			0.109			0.069
*p*			< 0.0001			0.0002			0.0046

* *p* < 0.05; ** *p* < 0.01; *** *p* < 0.001.

#### 3.1.2. Cognitive Failures as the Outcome

As shown in Table 2, of the variables in the model, only job demands was a significant predictor of cognitive failures. It remained a significant predictor after adjustment for detachment. Detachment was not a significant predictor of cognitive failures, and the bias corrected confidence intervals for the indirect effect includes zero (95% CI = −0.0459 − 0.0688); the results indicate that psychological detachment did not mediate the relationship between job demands and cognitive failures. In total, the model explained about 14% of the explained variance.

#### 3.1.3. Satisfaction with Life as the Outcome

Finally, as seen in Table 2, gender was the only control variable significantly related to satisfaction with life. In total, the model explained about 10% of the variance in the outcome. Both job demands and attachment were significant predictors of satisfaction with life, but after adjustment for detachment, job demands no longer had a significant association with life satisfaction. This impression of complete mediation was affirmed by the test of the indirect effect, in that the confidence interval did not include zero (95% CI = −0.2828 – (−0.0023)). This result thus aligns with the study hypothesis. Psychological detachment appeared to completely mediate the relationship between job demands and satisfaction with life. 

#### 3.1.4. Additional Control for Negative Affectivity

The supplementary analyses that included negative affectivity as an additional control variable led to similar conclusions for stress and cognitive failures. With satisfaction with life as the outcome variable, however, the degree of mediation no longer proved to be significant (95% CI = −0.2460 − 0.0123). Negative affectivity was a significant predictor in this analysis (B = −0.797, SE_B_ = 0.149, t = −5.340, *p* = 0.0000), whereas the direct effect of detachment on satisfaction with life (B = 0.217, SE_B_ = 0.123, t = 1.761, *p* = 0.0800) and the total effect of job demands on satisfaction with life (B = −0.096, SE_B_ = 0.158, t = −0.606, *p* = 0.5456) were no longer significant when it was included. Gender remained a significant predictor (B = 0.493, SE_B_ = 0.166, t = 2.967, *p* = 0.0035). 

### 3.2. Test of Moderation

Standard hierarchal regression analysis was used to test for moderation. The same control variables from the mediation analyses were entered in step one, giving similar coefficients as those from the mediation analyses. Job demands and detachment were entered in the second step. Finally, the Demands × Detachment interaction term was entered in the third and final step. Analyses were conducted separately for each outcome variable, and the results from the final step from each analysis are given in Table 3. To avoid problems with multicollinearity, those predictors also represented in the interaction term were mean centered before each analysis [30]. The variance inflation factors and tolerance values were examined for each analysis, but none of them indicated a problem with multicollinearity. We conclude the presentation of results by noting differences found when negative affectivity was included in the model.

#### 3.2.1. Perceived Stress as the Outcome

As shown in Table 3, detachment and job demands both showed significant main effects in the final step of the moderation analysis, with perceived stress as the outcome. The interaction term was not a significant predictor of perceived stress. Comparison of R^2^ for this model and R^2^ for the model without the interaction term (see Table 2) indicates that the interaction term contributed only negligibly to explained variance. In line with the study hypothesis, detachment did not moderate the relationship between job demands and stress. 

#### 3.2.2. Cognitive Failures as the Outcome

Job demands also showed a significant main effect in the final step of the moderation analysis, with cognitive failures as the outcome. Again, the interaction of demands and detachment was not significant and contributed little to the explained variance. In line with the study hypothesis, detachment did not moderate the relationship between job demands and cognitive failures.

**Table 3:** Results from the final step of the regressions analyses with psychological detachment moderating the relationship between job demands and outcome variables.

	Perceived stress			Cognitive failures			Satisfaction with life		
	B	SE_B_	t	B	SE_B_	t	B	SE_B_	t
**Control variables**									
Gender	0.354	0.090	3.917 ***	0.010	0.074	0.136	0.430	0.183	2.357 *
Deadline	−0.107	0.090	−1.193	0.54	0.073	0.733	0.114	0.181	0.630
Academic work weekends	−0.011	0.013	−0.894	−0.007	0.010	˗0.669	0.004	0.026	0.144
Job Demands	0.388	0.087	4.475 ***	0.304	0.071	4.286 ***	−0.203	0.175	−1.163
Detachment	−0.175	0.068	−2.579 *	−0.017	0.055	−0.302	0.288	0.137	2.108 *
Job demands × Detachment	0.013	0.085	0.154	0.009	0.070	0.126	0.112	0.172	0.650
*Model* *Summary*									
*R^2 ^*			0.337			0.136			0.099
*(R^2^adj)*			0.313			0.104			0.066
*p*			0.878			0.900			0.517

* *p* < 0.05; ** *p* < 0.01; *** *p* < 0.001.

#### 3.2.3. Satisfaction with Life as the Outcome

Detachment showed a significant main effect in the final step of the moderation analysis with satisfaction with life as the outcome. Again, the interaction effect was not significant, and the interaction term did not contribute to the explained variance. In line with the study hypothesis, detachment did not moderate the relationship between job demands and satisfaction with life.

#### 3.2.4. Additional Control for Negative Affectivity

The analyses that included negative affectivity as a control variable gave essentially similar results. We did not find evidence of a moderating effect of detachment for any of the outcome variables. For satisfaction with life, however, detachment no longer had a significant main effect (B = 0.225 SE_B_ = 0.127, t = 1.768, *p* = 0.079).

### 3.3. Discussion

The present study investigated the mediating *versus* moderating role of psychological detachment in the relationship between job stressors and psychological strain and well-being in a sample of advanced professional students. Psychological detachment was found to partially mediate the relationship between job demands and perceived stress and to completely mediate the relationship between job demands and satisfaction with life. Psychological detachment did not mediate the relationship between job demands and cognitive failures. More importantly here, psychological detachment did not moderate the relationship between job demands and any of the outcomes. 

The present study criticized the previously mentioned moderation studies for the small amounts of explained variance seen by their models and saw this as a reason to question their model fit. Concerning this, one could argue that interaction effects are harder to discover than main effects, but the estimates of variance explained and the associated *p*-values are what the authors of the critically assessed articles have provided in support of their moderation models, all the while ignoring evidence suggesting that a mediation model should be tested. Even if the true effect sizes were underestimated here, it would not change the fact that the results make a valuable contribution by finding differential evidence for the different models—no other study has yet attempted this.

The previously mentioned studies of psychological detachment as mediator or moderator [11,12,13,14] used somewhat different measures of job stressors and psychological strain, but all used the same measure of psychological detachment. The detachment measure was also presented to the participants in the same way, regardless of whether mediation or moderation was studied. None of those studies, however, raised the question of an alternative model, even when the job stressors investigated were significantly related to detachment (*i.e*., [13,14]) or mediation by detachment was inherent in the characteristics of the stressor (*i.e*., [14]). The present study did consider both mediation and moderation models and, in comparing the two models, found stronger evidence for mediation with the given set of job stressor and outcomes. 

When controlling for negative affectivity, detachment no longer significantly mediated the relationship between job stressors and satisfaction with life. Negative affectivity is an individual difference often controlled for in this research area. According to Sonnentag [4], it has been found to share about 10% of the variance with psychological detachment. Negative affectivity may influence the experience of detachment, demands and outcomes, as the correlational data in this study and others clearly suggest. Of the previous moderation studies mentioned, the one with the strongest moderation by detachment [14] did not adjust for negative affectivity. This clouds the view of detachment as a moderator of the relationship between work-family conflict and psychological strain in that study. Moreno-Jiménez, Rodríguez-Muñoz *et al*. [12] and Sonnentag, Binnewies *et al*. [13] did control for negative affectivity. As discussed by Spector *et al*. [27], however, adjustment for negative affectivity is problematic; it removes all variance shared with negative affectivity, regardless of why negative affectivity correlates with other variables. They also claim that negative affectivity could in fact play a substantive role in the job stress process. For example, people with a high degree of negative affectivity could, “by their behavior create or enact adverse circumstances [and], therefore, create job stressors for themselves” ([27], p. 88). In our model, the relationships affected were those involving the outcome variable, satisfaction with life. Conceivably, satisfaction with life is less sensitive to day-to-day strain than an outcome, like perceived stress, and it is explained to a greater degree by stable personal characteristics than by transient circumstances.

There may be alternative solutions to the mediation-moderation dilemma beyond the scope of this study. Perhaps there are other patterns that could be distinguished when psychological detachment works as a mediator and when it is a moderator. One possibility already alluded to is that the appropriateness of the model depends on the outcome measured, as well as the characteristics of the stressor, as with the results for satisfaction with life. There has also been some evidence of differences in day level detachment *versus* detachment over time [3]. Perhaps day level detachment mediates a relationship between stressors measured at the given time and the strain reported in conjunction with those stressors, whereas detachment measured over time is better approached as a moderator. Furthermore, the detachment scale asks participants about their experience of detachment in general, during their free time after work on weekdays. However, it can also be adapted to specify a specific time frame (e.g., [31]). The five studies discussed previously have used the general approach. A question arises whether the scale measures a stable disposition *versus* the effects of demands. Perhaps an adjustment of the scale items or instructions is needed when testing for mediation *versus* moderation. 

#### 3.3.1. Limitations of the Study

The present study has several limitations. The use of a convenience sample constrains generalization. The use of a cross-sectional design disallows causal statements. Nonetheless, we think the sample and the study design were suited to the intention of the study, to address a specific theoretical issue rather than generate estimates for broad generalization. We note that survey studies of employed individuals often use similar sampling methods, which involve approaching a convenient company or set of companies to enlist the participation of employees. This was the case for each of the previous studies we have cited, which were concerned with the mediating or moderating role of detachment.

The study’s choice of occupational model was that of advanced professional students, not employed individuals, and this might also be criticized. This is, however, not necessarily a major limitation of the study, as the students we surveyed were well invested in their fields, as reflected in their having completed at least one year of challenging studies, all of which had a practical focus and an orientation towards a future profession. It could also be argued that the Recovery Experience Questionnaire was initially designed for use in samples of employees who had well-defined periods when they were not “at work.” However, this does not appear to be a requirement of the scale, as it measures the perceived level of detachment and does not specify the extent or characteristics of the leisure time or activities during which detachment may be experienced.

Our sampling procedures did, however, entail the risk of self-selection bias. It is possible that those students who were experiencing the most strain at the time were also less likely to take time to participate. This may be the reason that the outcome measures had somewhat limited variability. Participants tended to report low values of stress and cognitive failures (M_PSS_ = 1.82, M_CFQ_ = 1.79, on a 0–4 scale) and high values of satisfaction with life (M_SWLS_ = 4.83, on a 1–7 scale), in combination with small standard deviations (SD_PSS_ = 0.66, SD_CFQ_ = 0.47, SD_SWLS_ = 1.14). Restriction of range makes it more difficult to assess correlations with other variables, and this may explain the weaker results concerning cognitive failures and satisfaction with life compared to perceived stress. 

Finally, the use of only self-report measures may have increased the risk of common method bias. We did, however, check for bias by negative affectivity in all models, and we only used well-established measures with previously demonstrated criterion validity and high internal consistency. These features of measurement and analysis should bolster confidence in the validity of our findings.

## 4. Conclusions

In this paper, we have indicated plausible reasons for some of the uncertainty regarding the role of psychological detachment from work as a mediator *versus* a moderator of the relationship between job stressors and strain. In doing this, we have pointed out some quite specific sources of ambiguity in the extant literature, and we have provided new empirical findings that address sources of uncertainty. Our results contribute to a view of psychological detachment as less an individual capability than a variable sensitive to the imposition of demands in the given context. Further research can address the additional possibility we have mentioned with regard to the measurement of detachment as an individual capability *versus* a feature of the conflict between work and other domains of life, and it can do so using additional measures of strain and well-being. The present results also encourage further consideration of how the possibilities for detachment change when work demands intrude into contexts that otherwise would provide relief for recovery, such as the home and outdoor recreational environments (*cf*. [32,33]). This intrusion of work into contexts normally used for recovery, and the resultant restriction of possibilities for detachment, may have significant implications for work-family conflict (*cf*. [34]). These will likely become issues of increasing importance as technological developments make it increasingly easy for many workers to bring their work with them almost anywhere and for employers to implicitly or explicitly encourage them to do so.
